# Evaluation of Blood Donation Awareness Level: A Cross-Sectional Study

**DOI:** 10.7759/cureus.47318

**Published:** 2023-10-19

**Authors:** Burcu Korkut

**Affiliations:** 1 Family Medicine, Karabük University, Faculty of Medicine, Karabük, TUR

**Keywords:** sociodemographic characteristics, knowledge level, blood donation, behavior, attitude

## Abstract

Introduction: This study aimed to evaluate the knowledge, attitudes, and behaviors of individuals regarding blood donation.

Methods: A questionnaire study was conducted with 644 individuals aged 18 and older who applied to a university hospital outpatient clinic between May 15, 2023, and August 15, 2023. Foreign nationals under the age of 18 were excluded from the study. The first six questions of the questionnaire were about sociodemographic characteristics. The other 28 questions were designed to assess knowledge, attitudes, and behaviors related to blood donation. The data obtained were analyzed with the SPSS 29 program. Categorical data were summarized as frequencies and percentages, and the relationship between variables was evaluated by the chi-square test, post hoc Bonferroni test, and Fisher’s Exact Test.

Results: Among the participants, 55% (n=354) were married, 57.1% (n=368) were female, 44.4% (n=286) were university graduates, and 24.8% (n=160) were between the ages of 18 and 24. There was a significant relationship between gender and age and individuals’ responses to the question about their previous blood donation status. Similarly, there was a significant relationship between the accuracy of answers and educational status and age in the question about the place of blood donation. There was a significant relationship between marital status, educational status, and age and responses to the question about those who were unable to donate blood or diseases that may be caused by blood donation.

Conclusion: Individuals’ knowledge, attitudes, and behaviors toward blood donation were found to be influenced by sociodemographic factors. Sociodemographic characteristics should be taken into account in activities to encourage blood donation and in the education of the community. It is believed that our study will shed light on future studies in this regard.

## Introduction

Blood donation is a practice that enables the transfer of blood from healthy individuals to those in need, and plays a life-saving role in many diseases and emergencies [1]. The demand for blood and blood products is constantly increasing, and to meet the need, regular and voluntary blood donation is of great importance [1]. In developed countries, 82% of blood donations come from voluntary blood donors, and donor blood is subjected to all screening tests. In developing countries, blood is obtained in different ways (such as blood for blood or forced transfusion) and only 50% are subjected to screening tests [2,3]. In addition, some complications such as weakness, fainting, and dizziness may occur during the blood donation process [4]. This condition is more common in young individuals, females, those who give blood for the first time, and those with low blood volume [4].

After all, blood donation is a vital process that should be explained to the public, misconceptions corrected, and questions answered [1]. Motivational initiatives should be implemented, and non-governmental organizations should be encouraged to focus on blood donation [5]. It is critical to continue blood donation research and to conduct larger-scale studies. This study aimed to assess the knowledge, attitudes, and behaviors of individuals about blood donation and to contribute to raising public awareness on this issue.

## Materials and methods

This cross-sectional study was conducted in Karabük University Faculty of Medicine, Department of Family Medicine between May 15, 2023 and August 15, 2023. A total of 644 patients aged 18 years and older who applied to the family medicine outpatient clinic for any reason and their relatives and to the Karabük University Training and Research Hospital Blood Donation Center for blood donation were included in the study. No sample selection was made in the study and the questionnaire consisting of 34 questions was applied to all participants with a face-to-face application technique. All voluntary participants were informed about the content and purpose of the study and the questionnaire, and their written informed consent was obtained. The inclusion criteria were determined as being 18 years of age or older and being willing to participate in the study with written consent. On the other hand, foreign nationals, pregnant women, those under 18 years of age, and illiterates were excluded from the study.

Within the scope of this study, a 34-question questionnaire form, which evaluates knowledge, attitudes, and behaviors related to blood donation, which was created by reviewing the current literature, was used (Appendices). The first six questions of the questionnaire were descriptive questions about age, gender, educational status, marital status, monthly income, and blood type. In this section, only age was an open-ended question. Information questions such as where blood donation can be done, the appropriate age, weight status for blood donors, complications that may arise from being a donor, and possible infectious diseases that may develop in blood donors were asked. In total, 13 questions were intended to measure the level of knowledge, and five of them were presented for evaluation as yes, no, no idea, and the remaining information questions were of multiple choices. The remaining 15 questions in the remaining part of the questionnaire were aimed at measuring attitudes and behaviors toward blood donation. It consisted of questions that questioned their behavior of donating blood to family members and/or strangers in case of need, the side effects they experienced, if any, when they donated blood before, and their suggestions for increasing blood donation. In question 28, participants were asked whether they had ever donated blood before. If their answer was no, they were asked to skip directly to the 34th question and complete the questionnaire; if their answer was yes, they were asked to end the questionnaire by answering the first 33 questions.

Approval for the study was obtained from the Karabük University Non-Interventional Clinical Research Ethics Committee (Number: E-77192459-050.99-81109 and Subject: 2021/ 717). All participants gave written informed consent.

The data obtained from the questionnaire were analyzed using SPSS 29 (IBM, Armonk, USA) statistical software. The chi-square test was applied to understand the distribution of the data and it was determined that it was compatible with non-parametric distribution. Categorical data were summarized as frequencies and percentages and the relationship between variables was evaluated by chi-square test, post hoc Bonferroni test, and Fisher’s Exact Test. A value of p<0.05 was considered statistically significant.

## Results

The sociodemographic characteristics of the participants in our study are listed in Table 1. Among the participants, 55% (n=354) were married, 57.1% (n=368) were female, 44.4% (n=286) were university graduates, 24.8% (n=160) were between 18 and 24 years of age, and 35.7% (n=230) had A Rh (+) blood type.

**Table 1:** Sociodemographic Characteristics of Participants

Marital status	n	%
Married	354	55
Single	290	45
Total	644	100
Gender		
Female	368	57.1
Male	276	42.9
Total	644	100
Education		
Elementary school	34	5.3
Secondary school	22	3.4
High school	200	31.1
University	286	44.4
Postgraduate	102	15.8
Total	644	100
Age Group		
18-24	160	24.8
25-30	122	18.9
31-38	82	12.7
39-45	112	17.4
46-54	72	11.2
55 and above	96	14.9
Total	644	100
Blood Type		
0 Rh (+)	194	30.1
A Rh (+)	230	35.7
B Rh (+)	74	11.5
AB Rh (+)	66	10.2
0 Rh (-)	20	3.1
A Rh (-)	20	3.1
B Rh (-)	6	.9
AB Rh (-)	12	1.9
Does not know	22	3.4
Total	644	100

The relationship between the sociodemographic characteristics of the participants and their answers to the question “Have you ever donated blood before?” is evaluated in Table 2. There was a statistically significant relationship between the participants’ responses (Yes or No) to the question and gender (p<0.05) and age (p<0.05). There was no statistically significant relationship between the participants’ responses (Yes or No) to the question and marital status (p>0.05) or educational status (p>0.05).

**Table 2:** Assessment of the Relationship Between Participants” Sociodemographic Characteristics and Their Responses to the Question “Have You Ever Donated Blood Before?” *p<0.05; chi-square test **p<0.05, chi-square test, Fisher’s exact test

Have you ever donated blood before?	Yes		No		p^*^
Marital status	N	%	n	%	0.382
Married	164	55.8	190	54.3	
Single	130	44.2	160	45.7	
Gender					0.0000^**^
Female	96	32.7	272	77.7	
Male	198	67.3	78	22.3	
Educational Status					0.860
Elementary school	14	4.8	20	5.7	
Secondary school	12	4.1	10	2.9	
High school	88	29.9	112	32	
University	132	44.9	154	44	
Postgraduate	48	16.3	54	15.4	
Age Group					0.000^**^
18-24	64	21.8	96	27.4	
25-30	66	22.4	56	16	
31-38	22	7.5	60	17.1	
39-45	44	15	68	19.4	
46-54	44	15	28	8	
55 and above	54	18.4	42	12	
Total	294		350		0.644

The relationship between the sociodemographic characteristics of the participants and their answers to the question “Where should blood be donated?” is shown in Table 3. There was no statistically significant relationship between the participants’ responses to the question and marital status (p>0.05) and gender (p>0.05). There was a statistically significant relationship between the participants’ responses to the question (to all Red Crescent branches throughout the country, to any hospital, to the Red Crescent mobile trucks, and to hospitals with blood donation centers) and their educational status (p<0.05). The participants were grouped by educational status, and the chi-square post hoc Bonferroni correction revealed that the high school and graduate groups differed (p<0.05) with the response “To hospitals with blood donation centers” compared to other education groups (p<0.05). There was a statistically significant relationship between the participants’ answers to the question “Where to donate blood?” and age (p<0.005). The participants were grouped by age, and the chi-square post hoc Bonferroni correction revealed that the 18-24 age group differed with the response “To hospitals with blood donation centers” and the 31-38 age group differed with the response “To any hospital” compared to other age groups (p<0.05).

**Table 3:** Evaluation of the Relationship Between the Sociodemographic Characteristics of the Participants and Their Responses to the Question “Where to Donate Blood?” *p<0.05; Chi-square Test; a,b: Bonferroni Test

Where to donate blood?	To all Red Crescent branches throughout the country		To any hospital		To the Red Crescent mobile trucks		To hospitals with blood donation centers		p^*^
Education	n	%	n	%	n	%	n	%	
Elementary school	8	4.1	4	4.8	11	6.4	11	5.6	0.001 (a: p=0.00067) (b: p=0.00014)
Secondary school	3	1.6	2	2.8	7	4.1	10	5.1	
High School^a^	53	27.5	29	34.9	39	22.7	79 ^a^	40.3	
University	90	46.6	38	45.8	77	44.8	81	41.3	
Postgraduate^b^	39	20.2	10	12	38	22.1	15 ^b^	7.7	
Age Group									
18-24^a^	44	22.8	15	18.1	39	22.7	62 ^a^	31.6	0.005 (a: p=0.0013) (b: p=0.0019)
25-30	43	22.3	11	13.3	38	22.1	30	15.3	
31-38^b^	18	9.3	19	22.9	20	11.6	25 ^b^	12.8	
39-45	37	19.2	14	16.9	37	21.5	24	12.2	
46-54	22	11.4	9	10.8	23	13.4	18	9.2	
55 and above	29	15	15	18.1	15	8.7	37	18.9	
Total	193		83		172		196		644

The relationship between the sociodemographic characteristics of the participants and the answers to the question “Who cannot donate blood?” is shown in Table 4. There was no statistically significant relationship between the participants’ responses to the question and gender (p>0.05). Chi-square and post hoc Bonferroni correction revealed a statistically significant relationship between the participants’ responses to the question (those with contagious diseases and those who have had any surgery) and marital status (p<0.05). There was a statistically significant relationship between the participants’ responses to the question (those with contagious diseases, those on medication, and those on aspirin) and educational status (p<0.05).

**Table 4:** Evaluation of the Relationship Between the Sociodemographic Characteristics of the Participants and Their Responses to the Question “Who Cannot Donate Blood?” *p<0.05; Chi-square Test; a,b,c,d: Bonferroni Test

Who cannot donate blood?	Those with any contagious disease		Those on medication		Those with diabetes		Those with disabilities		Those on aspirin		Those who have had surgery		Those under 18 years of age		Smokers		Other		p^*^
Marital Status	n	%	n	%	n	%	n	%	n	%	n	%	n	%	n	%	n	%	0.000 (a: p=0,0019), (b: p=0,0009)
Married^a^	103^a^	66	48	45.3	46	45.5	24	80	54	68.4	7	25.9	57	53.8	9	37.5	6	40	
Single^b^	53	34	58	54.7	55	54.5	6	20	25	31.6	20 ^b^	74.1	49	47.7	15	62.5	9	60	
Gender																			
Female	83	53.2	63	59.4	61	60.4	17	56.7	39	49.4	21	77.8	62	58.5	14	58.3	8	53.3	0.388
Male	73	46.8	43	40.6	40	39.6	13	43.3	40	50.6	6	22.2	44	41.5	10	41.7	7	46.7	
Education																			
Elementary school	7	4.5	5	4.7	8	7.9	1	3.3	3	3.8	1	3.7	5	4.7	1	4.2	3	20	0.000 (a: p=0,00017), (b,c: p=0,00046), (d: p=0,00031)
Secondary school	1	0.6	4	3.8	6	5.9	1	3.3	0	0	0	0	4	3.8	2	8.3	4	26.7	
High School^a,b,c^	27^a^	17.3	48^b^	45.3	30	29.7	10	33.3	11^c^	13.9	19	70.4	40	37.7	11	45.8	4	26.7	
University	82	52.6	40	37.7	44	43.6	10	33.3	45	57	6	22.2	46	43.4	10	41.7	3	20	
Postgraduate^d^	39^d^	25	9	8.5	13	12.9	8	26.7	20	25.3	1	3.7	11	10.4	0	0	1	6.7	
Age Group																			
18-24^a,b^	20 ^a^	12.8	35	33	33	32.7	4	13.3	8	10.1	15^b^	55.6	27	25.5	12	50	6	40	0.005 (a: p=0,000063), (b: p=0,00014)
25-30	40	25.6	13	12.3	19	18.8	5	16.7	23	29.1	3	11.1	15	14.2	4	16.7	0	0	
31-38	17	10.9	16	15.1	11	10.9	2	6.7	12	15.2	0	0	20	18.9	1	4.2	3	20	
39-45	36	23.1	14	13.2	16	15.8	11	36.7	12	15.2	4	14.8	14	11.9	3	12.5	2	13.3	
46-54	20	12.8	7	6.6	14	13.9	4	13.3	10	12.7	2	7.4	10	9.4	3	12.5	2	13.3	
55 and above	23	14.7	21	19.8	8	7.9	4	13.3	14	17.7	3	11.1	20	18.9	1	4.2	2	13.3	
Total	156		106		101		30		79		27		106		24		15		644

The participants were grouped by educational status, and chi-square post hoc Bonferroni correction revealed that the high school group differed with the response “those with contagious diseases, those on medication, and those on aspirin” and the graduate group differed with the response “those with contagious diseases” compared to the other education groups (p<0.05). There was a statistically significant relationship between the participants’ responses to the question (those with contagious diseases and those who have had any surgery) and age (p<0.05). The participants were grouped by age, and the chi-square post hoc Bonferroni correction revealed that the 18-24 age group differed from the other age groups with the response “those with contagious diseases and those who have had any surgery” (p<0.05).

The relationship between participants’ sociodemographic characteristics and answers to questions such as “Do you think blood donation may cause the following conditions or diseases?” is shown in Table 5. There was no statistically significant relationship between the participants’ responses to the question and gender (p>0.05). There was a statistically significant relationship between the participants’ responses to the question (AIDS and increased appetite) and marital status using chi-square post hoc analysis with Bonferroni correction (p<0.05). There was a statistically significant relationship between the participants’ responses to the question (AIDS) and educational status (p<0.05). The participants were grouped by educational status, and Bonferroni correction using the chi-square post hoc revealed that the postgraduate group differed with the response “AIDS” compared to other education groups (p<0.05).

**Table 5:** Evaluation of the Relationship Between the Sociodemographic Characteristics of the Participants and Their Responses to the Question “Do you think blood donation can cause the following conditions or diseases?” *p<0.05; Chi-square Test; a,b,c,d: Bonferroni Test

Do you think blood donation can cause the following conditions or diseases?	Weight loss		Infertility - Impotence - Early menopause		AIDS		Hepatitis		Allergy		Fever		Increased appetite		It causes none of them		p^*^
Marital Status	n	%	n	%	n	%	n	%	n	%	n	%	n	%	n	%	0.00001 (a:b: p=0.0019)
Married^a^	17	42.7	9	64.3	50 ^a^	72.5	33	45.2	34	60.7	30	47.6	9	28.1	172	57.1	
Single^b^	19	52.8	5	35.7	19	27.5	40	54.8	22	39.3	33	52.4	23^b^	71.9	129	42.9	
Gender																	
Female	21	58.3	6	42.9	36	52.2	45	61.6	30	53.6	39	61.9	19	59.4	172	57.1	0.843
Male	15	41.7	8	57.1	33	47.8	28	38.4	26	46.4	24	38.1	13	40.6	129	42.9	
Educational Status																	
Elementary school	1	2.8	2	14.3	3	4.3	2	2.7	3	5.4	4	6.3	2	6.3	17	5.6	0.001 (a:p=0.000004)
Secondary school	0	0	1	7.1	1	1.4	2	2.7	1	1.8	3	4.8	3	9.4	11	3.7	
High school	4	11.1	5	35.7	12	17.4	29	39.7	19	33.9	26	41.3	8	25	97	32.3	
University	21	58.3	6	42.9	29	42	35	47.9	25	44.6	27	42.9	11	34.4	132	43.9	
Postgraduate^a^	10	27.8	0	0	24^a^	34.8	5	6.8	8	14.3	3	4.8	8	25	44	14.6	
Age Group																	
18-24^a,b^	7	19.4	4	28.6	7^a^	10.1	25	34.2	10	17.9	24^b^	38.1	9	28.1	74	24.6	0.000 (a: p=0.00026) (b: p=0.00093) (c: p=0.00069)
25-30	11	30.6	1	7.1	18	26.1	13	17.8	7	12.5	10	15.9	10	31.3	52	17.3	
31-38 ^c^	3	8.3	0	0	13	18.8	2 ^c^	2.7	10	17.9	10	15.9	5	15.6	39	13	
39-45	10	27.8	1	7.1	17	24.6	9	12.3	10	17.9	7	11.1	5	15.6	53	17.6	
46-54	3	8.3	4	28.6	9	13	7	9.6	5	8.9	8	12.7	1	3.1	35	11.6	
55 and above	2	5.6	4	28.6	5	7.2	17	23.3	14	25	4	6.3	2	6.3	48	15.9	
Total	36		14		69		73		56		63		32		301		644

There was a statistically significant relationship between the participants’ responses to the question (AIDS, hepatitis, and fever) and age (p<0.05). The participants were grouped by age, and chi-square post hoc Bonferroni correction revealed that the 18-24 age group differed with the response “AIDS and Fever” and the 31-38 age group differed with the response “Hepatitis” compared to the other age groups (p<0.05).

## Discussion

This study was conducted with 644 people, of whom 57.1% were female, 55% were married and 24.8% were between the ages of 18-24. It was found that marital status and education level did not affect the participants’ previous blood donation status. Age and gender were found to be significant parameters in blood donation. Marital status and gender were not significant factors in indicating blood donation centers. However, there was a difference in the answer to this question according to age and education level. Education level, age, and marital status were significant in determining the diseases and conditions that prevented blood donation, while no difference was found due to gender. Similarly, marital status, age, and educational status were significant parameters for diseases that may develop as a result of blood donation, but no difference was found in this question depending on gender.

In this study, whether the participants had donated blood before was analyzed within the framework of sociodemographic characteristics. As a result, it was determined that age and gender parameters affected the blood donation behavior of the participants, but marital status and education level did not. Tadesse et al.’s study, which included multiple regression analysis with 556 healthcare workers in Ethiopia, found that 266 people donated blood at least once in their lives. In the same study, a significant relationship was found between factors affecting blood donation and being over 30 years old, being married, and having a high socio-cultural level [6]. Adıgüzel et al.’s study involving 446 university staff and students found that there was a significant relationship between genders in terms of blood donation status, with men donating blood at a higher rate [7]. Malako et al. conducted a study among hospital workers in Ethiopia, and very few of the participants reported having donated blood in their lifetime. It was also found that the practice of blood donation was more common among male participants [8]. In his research with the participation of 402 university students, Özpulat found that 14.7% of female students and 34.5% of male students had donated blood before [9]. Bourne et al.’s study conducted with 200 students at the Jamaica University of Technology found that although students had high knowledge and awareness about blood donation, they donated less blood [10]. In Altındiş et al.’s study conducted with 451 participants, it was revealed that 63% of the participants had never donated blood before [11]. In their study with 176 teacher participants, Hablemitoğlu et al. found that blood donation status and age were important predictors [12]. In his study of individuals living in rural areas of Thailand, Wiwanitkit found that education, especially depending on the school system, is an important factor affecting blood donation [13]. In Özbeşer et al.’s study conducted with 451 participants, it was determined that 44.2% of the participants had donated blood before, education level did not affect blood donation status, and men donated more blood than women [14]. In a study of 5353 donors in the Canary Islands, Romero Dominguez et al. found that women, teenagers, and college graduates tended to donate more [15]. Tscheulin and Lindenmeier’s study with 210 participants in Germany discovered that blood donors were young women or highly educated men [16]. In their study with 1055 primary care users in Brazil, Zucoloto et al. found that women, young people, and people with low socioeconomic and educational levels were less likely to donate blood [17]. When the results obtained in this study are compared with some studies in the literature, it is thought that the sociodemographic characteristics that affect blood donation behavior differ according to sample size, and ethnic and cultural characteristics.

In this study, it was determined that marital status and gender parameters were not significant in determining the places where participants could donate blood, but age and education level were significant. Similarly, as per Marantidou et al.’s study, at the event where 1600 blood donors were present in Greece, the participants stated that they could donate blood at any hospital in Greece [18]. Özpulat stated that 84% of the students donated blood in Red Crescent centers and 9.9% in public hospitals [9]. Tulunay, in his research with 2281 bank employees, stated that 39.1% of the participants made their first blood donation in the hospital, and the most blood donation was in the hospital. It was determined that the intended place was the Red Crescent Blood Center (53.8%) [19]. It is thought that especially age and education parameters are effective in choosing Red Crescent and hospitals as blood donation centers.

In this study, it was determined that education level, age, and marital status were significant in determining the diseases and conditions that prevent blood donation, but the gender parameter was not significant. It was also found that there was a significant relationship between the educational level of the participants and the responses of those with infectious diseases, those using medication, and those using aspirin. Saleh et al. similarly found that factors preventing individuals from donating blood were the donor’s medical conditions and nutritional deficiencies [20]. Oh et al. found in their study that people using low doses of aspirin (81 mg) could donate blood as long as their coagulation status was not significantly impaired [21]. Consistent with findings from comparable studies, health problems may be considered an important factor preventing individuals from donating blood.

In this study, it was determined that education level, age, and marital status were significant in determining the diseases and conditions that prevent blood donation, and there was no significant difference according to gender. Similarly, it was determined that the parameters of marital status, age, and education level were significant in diseases that may develop as a result of blood donation, but there was no difference according to gender. In their study with 86 people who gave blood samples before experiencing symptoms of the disease and a matched 256 control group, Kokkonen et al. found that cytokines, cytokine-related factors, and chemokines were elevated in donors before the onset of rheumatoid arthritis (RA) [22]. Olaiya et al. found that 52.4% of blood donors were concerned about HIV and/or hepatitis infection. The study found that many donors have misconceptions about the risks of donating blood, such as weight loss, sexual failure, and high blood pressure [23]. Özbeşer et al. found that 19.7% of participants were afraid of contracting AIDS after donating blood [14]. In the study conducted by Yaşar et al. with 100 physicians, it was found that 7% of donors avoided donating blood due to fear of contracting an infectious disease [24]. Altındiş et al. found that 34.1% of participants experienced fear of infection when donating blood [11]. Ryhan et al. in their study where 35,256 donors donated blood, it was determined that 1928 donors experienced negative effects and age and gender were effective in this effect [25]. The results obtained in this study and similar studies suggest that the responses to diseases that blood donation may cause, education level, age, and marital status have a positive impact on individuals’ awareness.

Limitations of the study: It is a single-centered study, thus the results cannot be generalized due to this situation, the validity and reliability of the survey questions in Turkish have not been conducted, and the Cronbach alpha number has not been calculated.

## Conclusions

This study revealed that knowledge, attitudes, and behaviors toward blood donation are affected by sociodemographic characteristics. In addition, it was seen that there was a significant lack of information in society about where to donate blood, who can be a blood donor, and complications that may occur due to blood donation, and awareness was raised. In order to increase blood donation and provide motivation in this regard, the public can be encouraged to donate blood, with healthcare professionals taking the lead in donating blood. Among the patients followed in primary care, those who are eligible to be donors can be supported by their family physicians to become donors. Additionally, increasing the number of mobile devices can positively affect blood donation. It is thought that the research can contribute to the studies on this subject and be a guide in this regard.

## Appendices

1. How old are you?

Your response

2. What is your gender?

•       Woman

•       Male

3. Your education status:

•       Illiterate

•       Literate

•       Primary school graduate

•       Secondary school graduate

•       High school graduate

•       High school graduate/university graduate

•       Master’s degree/PhD

4. Your marital status:

•       Married

•       Single

•       Widowed/Divorced

5. How much do you earn per month?

•       Income more than expenditure

•       Income equal to expenditure

•       Income less than expenditure

6. Indicate your blood type

•       0 Rh (+)

•       A Rh (+)

•       B Rh (+)

•       AB Rh (+)

•       0 Rh (-)

•       A Rh (-)

•       B Rh (-)

•       AB Rh (-)

•       I don't know

7. How can you get blood when you or someone close to you needs blood in a remote location?

•       Government pays

•       Red Crescent

•       I buy with money

•       Volunteer citizens welcome

8. Where can blood donations be made? (More than one option can be selected)

•       All Red Crescent branches across the country

•       Any hospital

•       To the mobile Red Crescent carts

•       Hospitals with blood donation centers

9. Do you think there is less need for common blood types?

•       Yes

•       No.

•       No opinion

10. Which age group can donate blood?

•       Healthy individuals aged 10-65 years

•       Healthy individuals aged 18-30 years

•       Healthy individuals aged 18-65

•       Healthy individuals aged 21-65 years

•       Healthy individuals of any age can give blood

•       No opinion

11. How much do you have to weigh to donate blood?

•       Over 40 kilos

•       Over 50 kilos

•       Over 60 kilos

•       Healthy individuals of any weight can give blood

•       No opinion

12. Who cannot donate blood? (More than one option can be selected)

•       Those with any infectious disease

•       Medication users

•       People with diabetes

•       Citizens with disabilities

•       Aspirin users

•       Those who have undergone any surgery

•       Under 18 years of age

•       Smokers

•       Other

13. Does blood donation have an effect on the person giving the blood? (More than one option can be marked)

•       No

•       It causes fatigue

•       Makes weight loss

•       Weight gain

•       Stimulates appetite

•       Addictive

•       Other

14. Would you donate blood if a relative or acquaintance needed blood in an emergency?

•       Yes

•       No.

•       No opinion

15. Would you donate blood if someone you don't know needed blood in an emergency?

•       Yes

•       No.

•       No opinion

16. Do you think blood donation can cause the following conditions or diseases? (More than one option can be selected)

•       Weight loss

•       Infertility

•       Impotence

•       AIDS

•       Hepatitis

•       Allergy

•       Fire

•       Early menopause

•       Stimulates appetite

•       It doesn't cause any of them

17. Have you ever received blood or blood products?

•       Yes

•       No.

•       I don't know

18. If you have been given blood or blood products before, what is the source? (More than one option can be selected)

•       Blood bank

•       Familiar

•       Soldier

•       Volunteer

•       We bought it with money

•       I don't know

•       I didn't take any blood products

19. Do you think the amount of blood donation in Turkey is sufficient?

•       Yes

•       No.

•       No opinion

20. Are there any known benefits of donating blood?

•       Yes

•       No.

•       No opinion

21. How many people can benefit from the blood donated by one person?

•       One person

•       More than one

•       No opinion

22. Do you pay attention to announcements about blood donation in public places (subway, hospital, dormitory, etc.)?

•       Yes

•       No.

•       Never heard of it

23. Would you recommend your relatives to donate blood?

•       Yes

•       No.

•       No opinion

24. In which way do you think blood and blood products are mostly supplied in Turkey?

•       Volunteer citizens

•       With money

•       Patient relatives

•       Red Crescent

•       Other

25. How do you think blood stocks should be met in our country?

•       Volunteer citizens

•       With money

•       Patient relatives

•       Red Crescent

•       Other

26. What should be done to encourage blood donation? (more than one option can be selected)

•       Money should be given

•       Blood tests should be done

•       Hygiene must be absolutely sure

•       Offer a meal after the donation

•       The blood donation assessment form should not have too many questions

•       Other

27. Is there any medicine that can be given to a patient in need of blood in case blood is not available?

•       Yes

•       No.

•       No opinion

28. Have you ever donated blood before?

•       Yes (continue with the survey)

•       No (skip to the last question)

29. How many times have you donated blood before?

•       1-5

•       6-10

•       More than 10

30. If you have donated blood more than once, how often?

•       Less than one per year

•       1-3 times a year

•       More than 3 times a year

31. What is the most important reason why you donate blood?

•       No specific reason

•       It's a good deed

•       It is a civic duty

•       Because my relative needed it.

•       To increase our belief that we can find blood when we need it

•       Because I think it's good for my body

•       Other

32. Have you experienced any side effects from donating blood?

•       Yes

•       No.

•       No opinion

33. Where was the place where you donated blood? (More than one option can be selected)

•       Red Crescent

•       Hospital blood bank

•       Mobile blood donation cart

34. If you have not donated blood before, what is the reason? (More than one option can be selected)

•       Because I'm afraid of needles.

•       Because I'm afraid of catching a contagious disease.

•       Because I don't trust health personnel

•       I do not feel healthy enough to give blood

•       I do not find it religiously correct

•       I never thought about giving blood

•       Because my parents wouldn't let me

•       Because I'm on medication

•       Never wanted

•       Other
